# Gastric Aspiration and Its Role in Airway Inflammation

**DOI:** 10.2174/1874306401812010001

**Published:** 2018-01-23

**Authors:** EB Hunt, A Sullivan, J. Galvin, J. MacSharry, DM Murphy

**Affiliations:** 1 The Department of Respiratory Medicine, Cork University Hospital, Cork, Ireland; 2 The Health Research Board Clinical Research Facility, University College Cork, Cork, Ireland; 3 The APC Microbiome Institute, Schools of Medicine and Microbiology, University College Cork, Ireland

**Keywords:** Reflux, Medicine, Inflammation, Lung Injury, Epithelium, Cytokines

## Abstract

Gastro-Oesophageal Reflux (GOR) has been associated with chronic airway diseases while the passage of foreign matter into airways and lungs through aspiration has the potential to initiate a wide spectrum of pulmonary disorders. The clinical syndrome resulting from such aspiration will depend both on the quantity and nature of the aspirate as well as the individual host response. Aspiration of gastric fluids may cause damage to airway epithelium, not only because acidity is toxic to bronchial epithelial cells but also due to the effect of digestive enzymes such as pepsin and bile salts. Experimental models have shown that direct instillation of these factors to airways epithelia cause damage with a consequential inflammatory response. The pathophysiology of these responses is gradually being dissected, with better understanding of acute gastric aspiration injury, a major cause of acute lung injury, providing opportunities for therapeutic intervention and potentially, ultimately, improved understanding of the chronic airway response to aspiration. Ultimately, clarification of the inflammatory pathways which are related to micro-aspiration *via* pepsin and bile acid salts may eventually progress to pharmacological intervention and surgical studies to assess the clinical benefits of such therapies in driving symptom improvement or reducing disease progression.

## 1 BACKGROUND

Gastro-Oesophageal Reflux Disease (GORD) affects approximately 20% of the population of the western world [1]. The possible relationship between GORD and airways disorders has long been postulated. While physicians generally acknowledge a link between oesophageal disorders and respiratory disease, this relationship is complex and may manifest in a variety of clinical ways. In particular, Gastro-Oesophageal Reflux (GOR) seems to be frequently present in patients with advanced lung diseases [2].

Gastro-oesophageal reflux is caused by stomach contents leaking into the oesophagus, which in turn has the potential to lead to aspiration of these contents into the adjoining respiratory tract. In healthy subjects, the amount of aspirated material is usually small and can be cleared by host defense mechanisms without consequence. However, if aspiration is to result in adverse clinical consequences, the nature of the evolving illness will be dependent on the nature and volume of the aspirated material, the temporal nature of the insult, and the elicited host responses [3]. Aspiration of large amounts of gastric acid will result in the induction of a chemical injury to the airways and lung parenchyma. The initial insult triggers a cascade of inflammatory responses with the recruitment of inflammatory cells and the release of various inflammatory mediators [4]. Large volume gastric acid aspiration may cause an acute lung injury, with diffuse alveolar damage and pneumonitis. In contrast, recurrent small volume aspiration during sleep appear may be without consequence or result in chronic and less severe forms of lung injury [3]. Damage to the airway epithelium from aspiration of gastric fluids may be due to the toxicity of its low pH, (with Song *et al.* having previously demonstrated normal airway pH to be 7.3 independent of airway size) [5]. *In vivo* and *in vitro* models have examined the effect of acid aspiration on lung injury and inflammation, using hydrochloric acid, with a pH ranging from 1 to 1.5. Davidson *et al.* crucially demonstrated that gastric particles (both acidified and non-acidified) were also found to contribute to lung injury [6]. It had previously been shown that neutrophil influx into the lungs is mediated *via* an interleukin-6 (IL-6) and IL-8 pathway with Sacco *et al*. demonstrating a relationship between acidity and aspiration, increased IL-8 levels and airway neutrophil counts in asthmatic patients [7].

In those individuals who aspirate, recurrent respiratory tract infections are a common consequence, with the ensuing development of bronchiectasis a possible end-stage manifestation. Normal inflammatory responses to infection exhibited by macrophages are blunted due to weakly acidic conditions [8]. In the Cystic Fibrosis (CF) pig model, loss of antimicrobial peptide activity causes a reduction in bacterial killing, as a direct consequence of a reduction in airway surface liquid pH [9]. Epithelial regulation of local immunity through secretion of chemoattractants such as IL-8 in response to infection has been postulated with Hackett *et al.* showing that a weakly acidic environment might blunt this response [10]. In a cohort of patients prone to micro-aspiration they investigated whether or not lipopolysaccharides (LPS)-induced expression of inflammatory/chemotactic proteins was modulated by an acidic environment [11]. In cells exposed for 4 hours at weakly acidic pH, LPS-induced cytokine expression was reduced. Expression was further reduced on 16 hours exposure to low pH, but this time a significant decrease in cell viability was also noted. The authors subsequently observed a significant reduction in LPS-induced cytokine production following weakly acidic shock treatment and subsequent prolonged exposure to endotoxin at normal pH, in turn potentially compromising the response to infection through the reduction in cytokine production and the potential for subsequent diminished immune cell recruitment.

Recent research has shown that microbiological growth from gastric juice occurred when gastric juice pH was >4 and that the gastric and airway microbiome compositions of people with CF who reflux are similar in profile. This novel data suggests the existence of an aero-digestive microbiome in CF, which may ultimately have clinical relevance. This host response to aspiration as well as its potential to induce changes in the pulmonary microbiome needs further investigation, with its importance only gradually gaining widespread recognition [12-14].

## 2 PEPSIN AND BILE ACIDS

The acidity of gastric fluids might not be the only potential mechanism of airways damage and a pro-inflammatory response to aspiration. It is possible that digestive enzymes such as pepsin and Bile Acids (BA) may also play roles as mediators of this airway response to aspiration injury.

Pepsin is stored as inactive pepsinogen in the chief cells of the gastric mucosa. It is a protease involved in the digestion of food, and its activity is acid-dependent. The conversion of pepsinogen to pepsin in the stomach starts slowly at pH 6 and reaches optimal activity between pH 1.5 to 2.5. Above pH 6.8, pepsin becomes inactive and above pH 7.5 it is fully inactive and irreversibly denatured [15]. In human gastric fluid, the pH ranges from 1.5 to 3, which allows optimal pepsin activity, and the concentration of pepsin varies from 0.5 to 1 mg/ml [16].

In recent years, there has been an increasing acceptance of measured pepsin within the airways as representing an important and reliable biomarker for gastric aspiration [17-19]. Detectable airway pepsin has been associated with broncho-pulmonary dysplasia in children and with post lung transplant allograft rejection [20, 21]. Clinical and scientific experimental studies are needed to delineate whether pepsin is solely a marker of aspiration, or whether these associations are in part due to the pathological actions of pepsin. Amplification of cell and tissue damage may be a result of the combined breakdown of protein by pepsin and independent acid damage. In oesophageal tissue of rabbits, increased tissue damage has been demonstrated with exposure to pepsin in acid as compared to the tissue damage caused by the action of acid administered in isolation [22]. The cytotoxicity and inflammation caused by a combination of acid and pepsin on airway epithelium has, to date not been fully defined.

Utilizing a bronchial epithelial cell model, Bathoorn *et al*. found pepsin to be both cytotoxic and to induce an inflammatory response in cells [23]. Pepsin-induced cytotoxicity to human bronchial epithelial (16HBE) cells is pH-dependent, with the greatest effect seen at the lowest acidity. Similarly, pepsin-mediated IL-6 and IL-8 release is optimized through lowering the pH of the media. *In vivo* studies have found that erosive lesions in esophageal epithelium exposed to pepsin are caused by the destruction of junctional molecules, with similar lesions described in the airways following gastric fluid aspiration [22].

Gastro-oesophageal reflux-derived bile acids have been detected in the Bronchoalveolar Lavage (BAL) fluid and sputum of patients with GOR (and co-existent respiratory conditions) at concentrations ranging from 0.4 μM up to 32 μM [24, 25]. A predisposition to pulmonary infection has been associated with Bile Acid (BA) aspiration in several studies [25-29], including infection with *Pseudomonas aeruginosa*, an important pathogen associated with several respiratory diseases. This is particularly relevant in the CF patient population, as the presence of pseudomonas in the CF airway is associated with increased morbidity and mortality [30, 31]. Bile acid aspiration has been linked with airways inflammation, with a correlation between alveolar neutrophils and interleukin-8 (IL-8) with BAL fluid BAs described in post- lung transplant and cystic fibrosis populations [25, 26, 29].

Bile acid aspiration has also been associated with increased BAL tumor necrosis factor alpha (TNF-α) in a rodent model of chronic aspiration, leading to downstream pro-inflammatory cascade [34]. However, the definitive mechanisms underpinning the impact of BA aspiration on lung inflammation and infection remain poorly elucidated. It is possible that the presence of BAs in the airways may trigger host factors, such as transcription factors, which in turn may modulate the ensuing immune response pathways. One potential target is hypoxia-inducible factor 1 (HIF-1). HIF-1 has been characterized to be an emerging master regulator in the host response to infection [32-35] and inflammation [36]. HIF-1 induces genes involved in the host immune response, such as nitric oxide, antimicrobial peptides, and several cytokines, including TNF-α, which have been demonstrated to play an important role in containing infection [37]. Legendre *et al.* proposed that the suppression of HIF-1 signaling by BAs may have a significant influence on the progression and outcome of respiratory disease. They further found that the effects of BAs on cytokine production could at least as important as bacterium-mediated responses [38].

The inflammatory cascade that results from direct caustic action of pH and the exposure to acidic gastric aspirate has been well established but what of the non-acidified gastric aspirate? There is growing evidence from animal studies for small (~10mm) non-acidified gastric particles (SNAPs). Studies using tracheal instillation of SNAPs in rats demonstrated an acute neutrophilic inflammatory response at 4-6 hours. The initial oedema seen in previous studies using the aspiration of dilute hydrochloric acid (HCL) to form an animal model, was however not seen [39]. Acute inflammatory markers including TNF-α, CXCL2 and CINC-1 were found to be elevated in the BAL from rats [40, 41]. It has been postulated that the presence of Monocyte Chemoattractant protein-1 (MCP-1/CCL2) may be responsible for the innate pulmonary inflammatory response and the development of granuloma. CCL2 is produced by numerous cells including vascular endothelial cells and alveolar type II pneumocytes. Mounting evidence suggests that CCL2 and its haematopoietic cell receptor CC Chemokine Receptor 2 (CCR2) are involved in inflammatory disorders of the lung. In animal models of allergic asthma, idiopathic pulmonary fibrosis (IPF), and bronchiolitis obliterans syndrome (BOS), CCL2 expression and protein production are increased and the disease process is attenuated by CCL2 immuno-neutralization. Postulated mechanisms of action include recruitment of regulatory and effector leukocytes; induction of fibroblast production of transforming growth factor-β (TGF-β) and pro-collagen; stimulation of histamine or leukotriene release from mast cells or basophils; and enhancement of Th2 polarization [42].

In truth, the severity of injury associated with gastric aspiration is as a result of a combination of the two insults, both acid and SNAP. In both rat and murine models, the severity of lung injury from a combined injury is more severe than for either SNAP or as a result of acid exposure alone [40, 43, 44]. Levels of albumin in BAL, which when raised, is associated with the loss of membrane integrity, are significantly higher in those rats exposed to the dual insult. There is in fact an observed synergistic response that may account for the increased severity of lung injury observed, one that is far greater than injury levels occurring with individual component injuries alone. Neutrophils (numbers) are dramatically increased in rodent BAL who have been subjected to a dual insult compared to acid or SNAP alone. These levels remain high 48 hours post aspiration, indicating leukocytic infiltration [40]. Levels of CINC-1 (rat homologue of IL-8), IL-10, CCL2 were also elevated in those exposed to dual insult compared to ACID or SNAP alone. In this model, IL-10 levels were the single best predictor of lung injury severity [40]. This finding is consistent with the interpretation that increases in this anti-inflammatory (down-modulatory) cytokine are important in protective responses against acute inflammation. Treatment with IL-10 reduces the influx of inflammatory cells and suppressed the expression of IL-6, TNFα and CXCL2. Upon exposure, NF-κB activation is one of the first pulmonary responses, and it occurs before the detection of any increase in pro-inflammatory cytokines [45]. Nuclear factor-κB controls the expression of some 200 target genes, many of which are involved in inflammation, such as adhesion molecules, interleukins, chemokines, acute phase response genes and cytokines [46]. In Li. et al’s study, treatment with exogenous IL-10 reduced NF-κB p65 expression, which might mediate the suppressive action of IL-10 on the formation of these pro-inflammatory cytokines and subsequent inflammation [47].

## 3 ACUTE LUNG INJURY

Inflammation related to aspiration and reflux not only plays an important role in chronic respiratory diseases such as asthma, COPD, IPF, Cystic Fibrosis, and in the post lung transplant population, it is also apparent that it plays a significant role in acute respiratory illness. Aspiration-induced lung injury often goes unrecognized in critically ill patients and it is postulated that it may account for a significant proportion of acute pulmonary dysfunction in this setting [39]. Despite this, aspiration is well recognized as an independent risk factor for the subsequent development of pneumonia or acute lung injury or acute respiratory distress syndrome (ALI/ARDS) [48]. The underlying mechanisms responsible for the progression to severe pulmonary inflammation with ensuing ALI/ARDS as a result of gastric aspiration are not fully understood. In a prospective study of critically ill patients, Metheny and colleagues found that in patients who were ventilated and tube fed for a minimum of 4 days that at least one aspiration event occurred in almost 90% of the patients [49]. The degree of lung injury as a consequence of gastric aspiration ranges from mild pneumonitis to severe, progressive respiratory failure with an associated significant detrimental impact on patient outcome. It has been recognized therefore that gastric aspiration may directly cause ALI and the more severe clinical phenotype, ARDS [39, 50, 52]. The clinical development of ALI/ARDS typically involves a sudden, severe pulmonary inflammatory injury with the loss of integrity of alveolar-capillary permeability. Hence, this injury may result in alveolar edema, loss of lung compliance, hypoxemia, and frequently is associated with multi-organ system failure [39, 42]. The economic and healthcare impact of ALI/ARDS is significant, with economic costs of 3.5 – 6 billion dollars and mortality levels of 30-40% reported in the United States [53, 54]. Furthermore, aspiration pneumonitis and ensuing ALI/ARDS is believed to account for up to 20% of all deaths attributable to anesthesia [55-57].

Animal experimental models of the acid component of gastric aspirates are frequently created by intra-tracheal instillation of HCL. Following aspiration of dilute HCL, lung injury in adult rats is characterized by a biphasic response. The early phase insult is mediated by stimulation of capsaicin sensitive neurons as well as the direct caustic actions of low pH on airway epithelium, and is followed by an acute neutrophilic inflammatory response at 4-6 hours [58]. The combination of these mechanisms leads to the loss of microvascular integrity, extravasation of fluid and protein into the airways and alveoli. The presence of fluid secondary to oedema may reduce airway compliance while the plasma proteins in the oedema fluid can directly interfere with alveolar surfactant function. Aspiration of low pH gastric contents is characterized by a neutrophilic airway influx. Levels of TNF–α and concentrations of several important chemotactic cytokines (chemokines) in BAL have also been found to be elevated following gastric aspiration [59, 60] Fig. (**1**). This includes elevated levels of the neutrophil chemotactic chemokine IL-8 in rabbits, or CXCL2 and cytokine induced neutrophil chemoattractant–1 (CINC-1) in rodents, during acid induced lung injury [40, 41, 61]. Levels of leukocyte derived oxidants and proteinases, including elastase have similarly been shown to be increased in acid-induced lung injury [62, 63]. Activation of complement is also described as important in the systemic response and the consequent injury that occurs following gastric aspiration [64].

Recruitment of neutrophils and release of reactive oxygen species are considered to be major pathogenic components driving ALI. However, NADPH oxidase, the major source of reactive oxygen species in activated phagocytes, can paradoxically limit inflammation and injury. NADPH oxidase generates the oxidative “burst” in PMNs, leading to the production of ROS (reactive oxygen species) and activation and release of PMN granular proteases [65-68] In addition to this enzyme’s critical host defense function, NADPH oxidase also regulates inflammation. In studies of lung inflammation induced by microbial-derived products, NADPH oxidase restrained lung inflammation by activation of Nrf2, a redox-sensitive anti-oxidative and anti-inflammatory transcription factor [66]. Experiments by Davidson *et al.* in mice models of ALI showed that NADPH oxidase activation leads to rapid generation of ROS and activation of PMN granular proteases responsible for killing invading pathogens [69]. While initial actions are clearly injurious, the study demonstrated that NADPH oxidase could also counterbalance these early pro-inflammatory and injurious events.

## 4 ANTI-INFLAMMATORY TREATMENT

Our knowledge of the pathogenesis of reflux mediated cell injury has exponentially expanded over the last number of years. However, despite an improved understanding of the mechanisms involved, this has not led to any new forms of treatment. Despite the acknowledgement that pepsin, bile salts, acidic and non-acidic reflux play interchangeable roles in the end pathology of aspiration, treatment options are still restricted to proton pump inhibitors (PPI) and surgical intervention, both of which have yielded mixed results. Understanding of the role played by PPIs comes from studies looking at their effect on acidic bile salts. Huo *et al.* demonstrated the omeprazole inhibits IL-8 secretion stimulated by exposure to acid and bile salts from esophageal epithelial cell [70]. They demonstrated that acidic bile salts activate the IL-8 promoter through nuclear factor (NF)- κB and activator protein (AP)-1 DNA binding sites, and that the omeprazole inhibits IL-8 production by blocking nuclear translocation of p65, as well as decreased binding of p65, c-jun and c-fos to the IL-8 promoter. These effects were independent of the effects on gastric acid secretion demonstrating a novel mechanism that might contribute to the beneficial effects of PPIs in the treatment of reflux related pulmonary inflammation. The caveat to this finding comes in research by Pauwels *et al.* examining the effect of aspirate of CF patients being treated with a PPI, that showed a significantly enhanced inflammatory effect (higher IL-8 production) on CF bronchial epithelial cells in culture. As chronic PPI treatment in CF may result in a paradoxically increased inflammatory effect in the airways, alternative anti-reflux therapies should be considered in CF. If uncontrolled GORD is present and medical management fails, fundoplication may have a role in preserving lung function and improving pulmonary status in a small, carefully selected patient group [71]. Interleukin-6 is one of the key mediators of fibrosis in chronic inflammation in animal models, and it may be that better understanding of these critical elements within the inflammatory cascade may yield pharmacogenic therapies in the years to come.

## CONCLUSION

Available clinical and experimental evidence points to a possible relationship between the progression of airways disease, pro-inflammatory processes and gastric aspiration. In order to progress this further it is now critical to initiate development and implementation of pharmacological or surgical interventions in the relevant patient groups, targeted at reducing gastric aspiration rates with objective measures of airways disease as the primary outcomes in adequately powered, controlled studies. Such studies will ultimately allow us to determine whether any causal relationship between aspiration and severity of airways disease does indeed exist.

## Figures and Tables

**Fig. (1):**
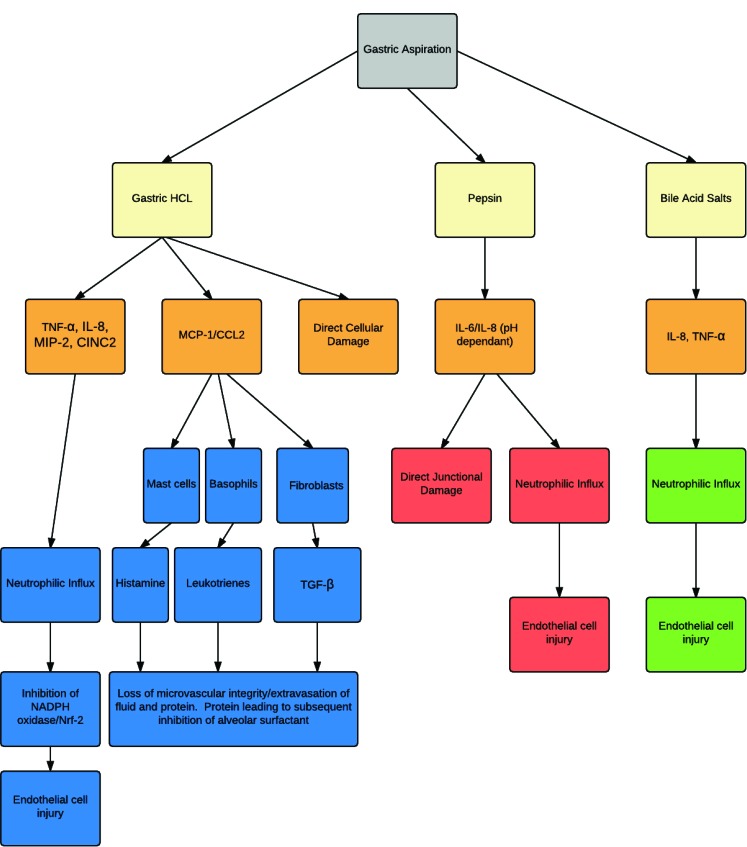
Gastric aspiration and the complex inflammatory cascade in airway epithelium and endothelium. Direct and indirect pathways when exposed to acid environment as well as the parallel pathways involving pepsin and bile salts, ultimately leading to endothelial damage, and acute and chronic lung injury. TNF- α (Tumour necrosis factor-α), IL-8 (Interleukin-8), MIP-2 (macrophage inflammatory protein), CINC2 (Cytokine-induced neutrophil chemoattractant), MCP (Monocyte chemotactic protein), CCL2 (chemokine (C-C motif) ligand), TGF-β (Transforming growth factor), NADPH (Nicotinamide adenine dinucleotide phosphate), and NFR-2 (Nuclear factor (erythroid-derived 2)-like 2).

## Data Availability

Not applicable.
